# Rectus sheath hematoma causing cecal perforation: a case report

**DOI:** 10.1093/jscr/rjaf550

**Published:** 2025-07-17

**Authors:** Abanoub Awad, Mitchell Meagher, Isaac Theerman, Jason Beckermann

**Affiliations:** Department of Surgery, Mayo Clinic Health System, 1400 Bellinger St. Eau Claire, WI 54703, United States; Department of Surgery, Mayo Clinic Health System, 1400 Bellinger St. Eau Claire, WI 54703, United States; Department of Surgery, Mayo Clinic Health System, 1400 Bellinger St. Eau Claire, WI 54703, United States; Department of Surgery, Mayo Clinic Health System, 1400 Bellinger St. Eau Claire, WI 54703, United States

**Keywords:** rectus sheath hematoma, cecal perforation, anticoagulation, case report, abdominal pain

## Abstract

Rectus sheath hematoma is a rare condition caused by bleeding from the epigastric arteries, with an incidence of 1.2–1.5 cases per year. We present a 49-year-old male with a smoking history and recent corona virus disease 2019 (COVID-19) infection who presented with symptoms of an upper respiratory infection and suspected venous thromboembolism. Imaging revealed bilateral pulmonary emboli and a left rectus sheath hematoma, which was initially managed conservatively. However, the patient’s condition worsened with a significant drop in hemoglobin and development of encephalopathy. Imaging showed an enlarging hematoma, leading to transfusion and selective embolization. On day 8, the patient developed generalized abdominal pain, and imaging confirmed a hollow viscus perforation. An exploratory laparotomy revealed cecal perforation due to mass effect from the hematoma. An ileocecectomy was performed.

## Introduction

Rectus sheath hematoma (RSH) is an uncommon clinical condition that occurs due to bleeding from epigastric arteries or their muscular branches within the rectus sheath [1, 2]. RSH can present as abdominal pain mimicking other intra-abdominal pathologies, such as appendicitis, cholecystitis, diverticulitis, or generalized peritonitis; additionally, the presence of ecchymosis can mimic other pathological processes such as pancreatitis (Grey Turner’s/Cullen’s sign) [3]. The incidence of RSH has been estimated at 1.2–1.5 cases/100 000 per year based on observations in the radiology literature, and the condition occurs three times more frequently in women than in men [4].

## Case report

We present a 49-year-old male with a 23-pack-year smoking history and prior COVID-19 infection 1 month before admission. His body mass index was 29 kg/m^2^. He presented to the Emergency Department with symptoms of an upper respiratory tract infection. Initial workup raised concern for venous thromboembolism (VTE) and pulmonary embolism (PE), supported by an elevated D-dimer level. A computed tomography (CT) of the chest confirmed bilateral pulmonary emboli. The patient was admitted, started on a heparin drip, and oxygen therapy.

**Figure 1:**
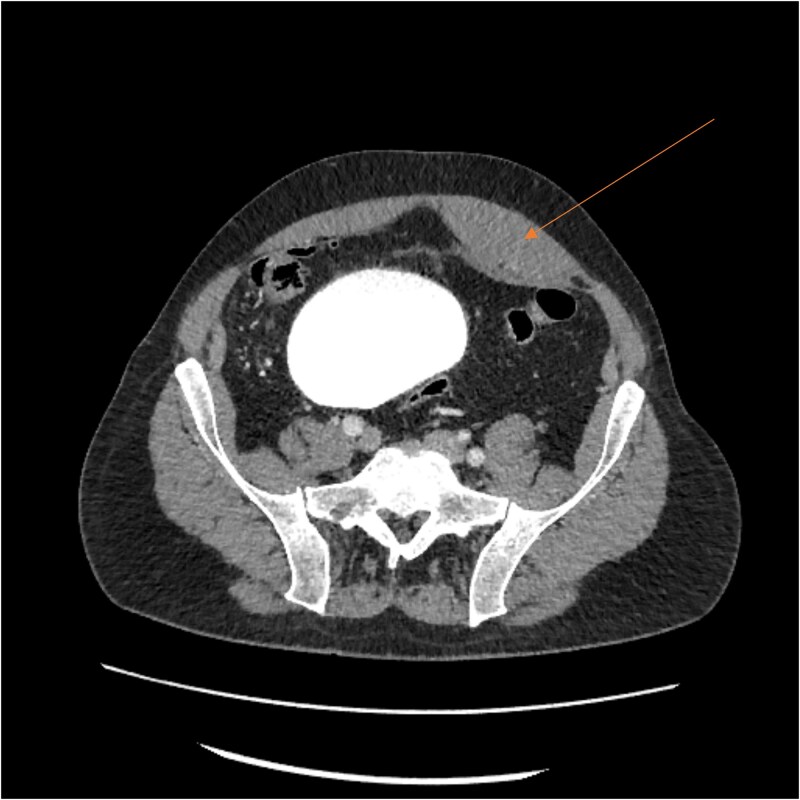
CT scan showing left RSH (arrow).

Laboratory results showed thrombocytopenia and erythrocytosis, with a hemoglobin level of 18.6 g/dl and platelets at 840 00/μl. A CT scan of the abdomen and pelvis was ordered to rule out malignancy or paraneoplastic syndrome causing the VTE (Fig. 1). The CT showed no lesions suspicious for malignancy but revealed a left RSH, managed conservatively.

**Figure 2:**
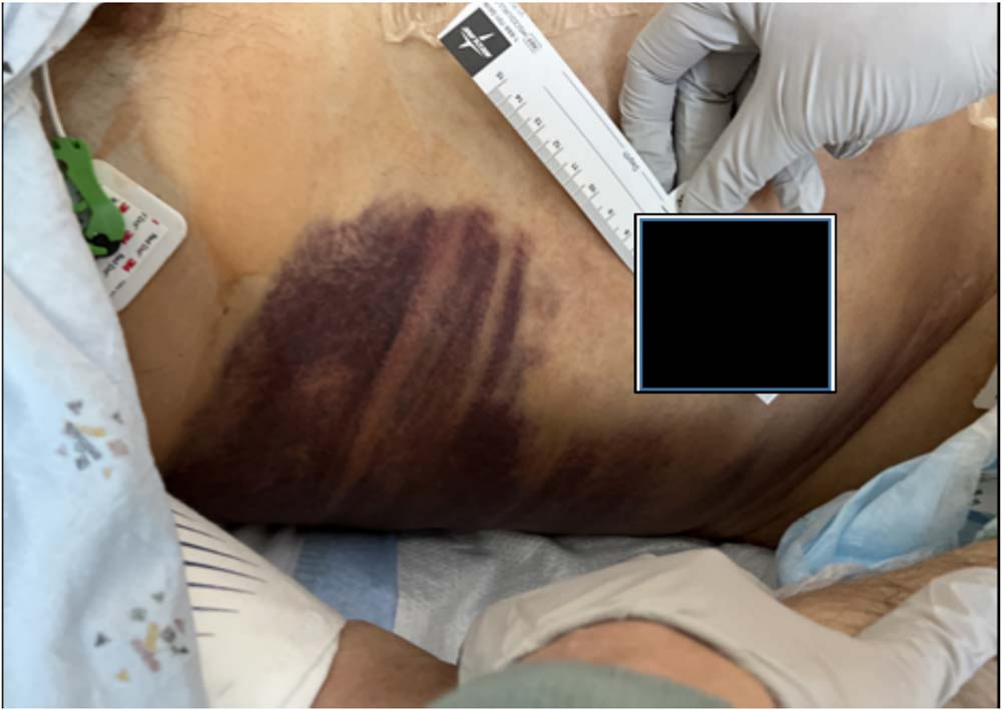
Grey Turner's sign.

On day 2, the patient was transitioned to warfarin with a Lovenox bridge and discharged to a subacute service for ongoing monitoring. While at home, his hemoglobin dropped progressively, but he declined to return for further investigation. Three days after discharge, the patient developed significant encephalopathy, requiring transfer back to the hospital. Upon arrival, he was hypotensive and lethargic, requiring intubation, phenylephrine boluses, norepinephrine infusion, and anticoagulant reversal with Kcentra. Labs showed a hemoglobin drop to 6.5 g/dl, creatinine increase to 5.5 mg/dl, with profound metabolic derangements of a combined respiratory and metabolic acidosis (pH = 6.58). Physical examination revealed Grey Turner’s sign (Fig. 2).

**Figure 3:**
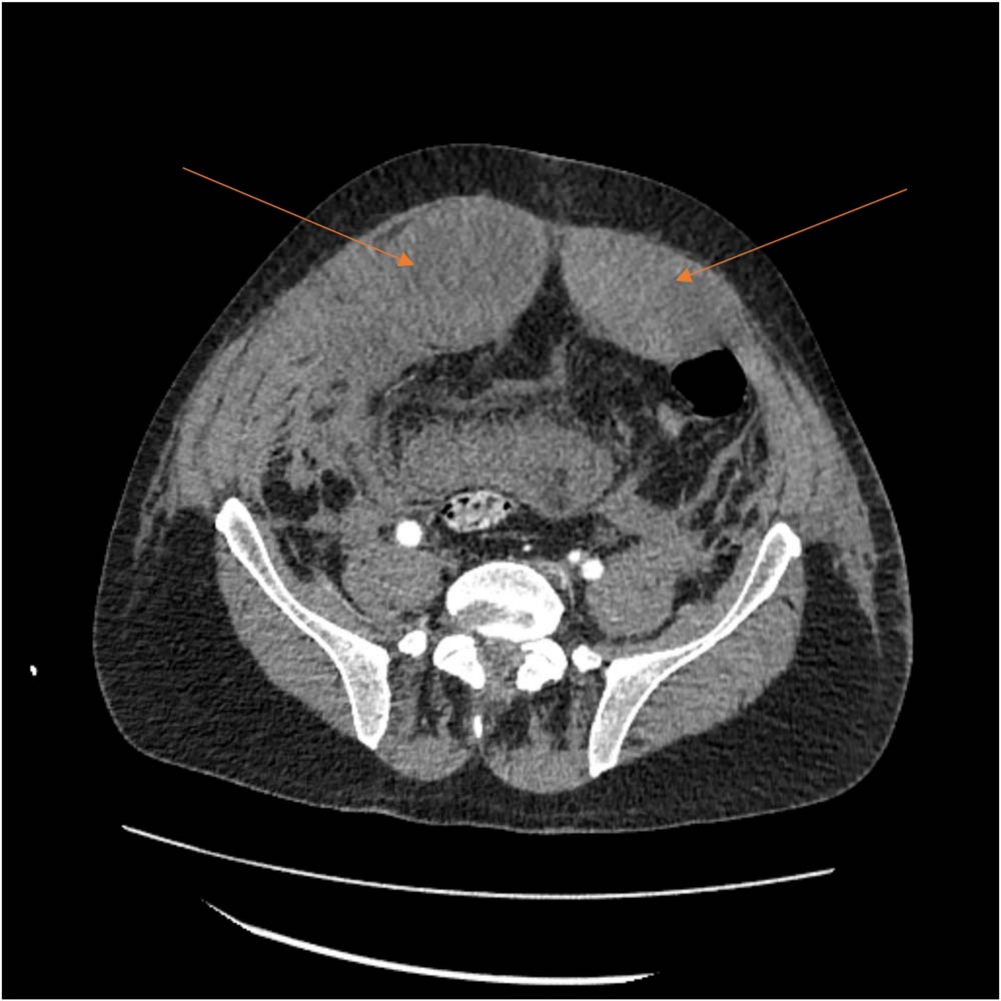
A follow-up CT scan showing progression of the RSH (arrows).

A massive transfusion protocol was initiated, and the patient received four units of packed red blood cells, six units of fresh frozen plasma, two units of platelets, and bicarbonate boluses. A CT abdomen and pelvis was performed to reassess the RSH, revealing an interval enlargement (15.3 × 10.7 × 20.4 cm) with a focal blush at the inferior portion, concerning for active extravasation (Fig. 3). The patient was hemodynamically stabilized and underwent inferior vena cava (IVC) filter placement once his hemoglobin reached 8 g/dl.

**Figure 4:**
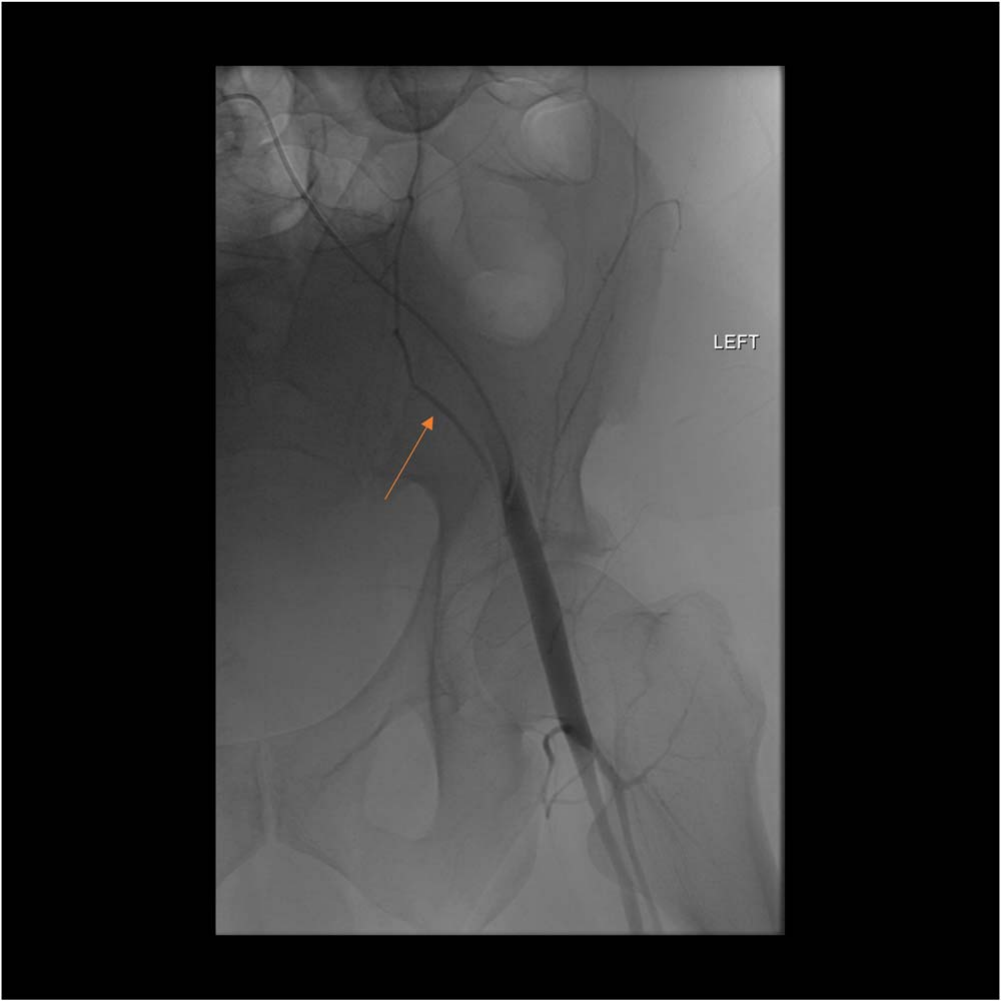
Angiogram of the left inferior epigastric artery (arrow) demonstrating no active bleeding.

A follow-up CT scan performed 2 days after placement of IVC filter and stopping anticoagulation showed further enlargement of the hematoma (17.5 × 14 × 25.7 cm) and ongoing bleeding. Interventional radiology performed bilateral inferior epigastric artery angiograms, which revealed no ongoing artery bleeding but truncation of the right inferior epigastric artery. Empiric Gelfoam embolization was performed on the left inferior epigastric artery (Fig. 4).

**Figure 5:**
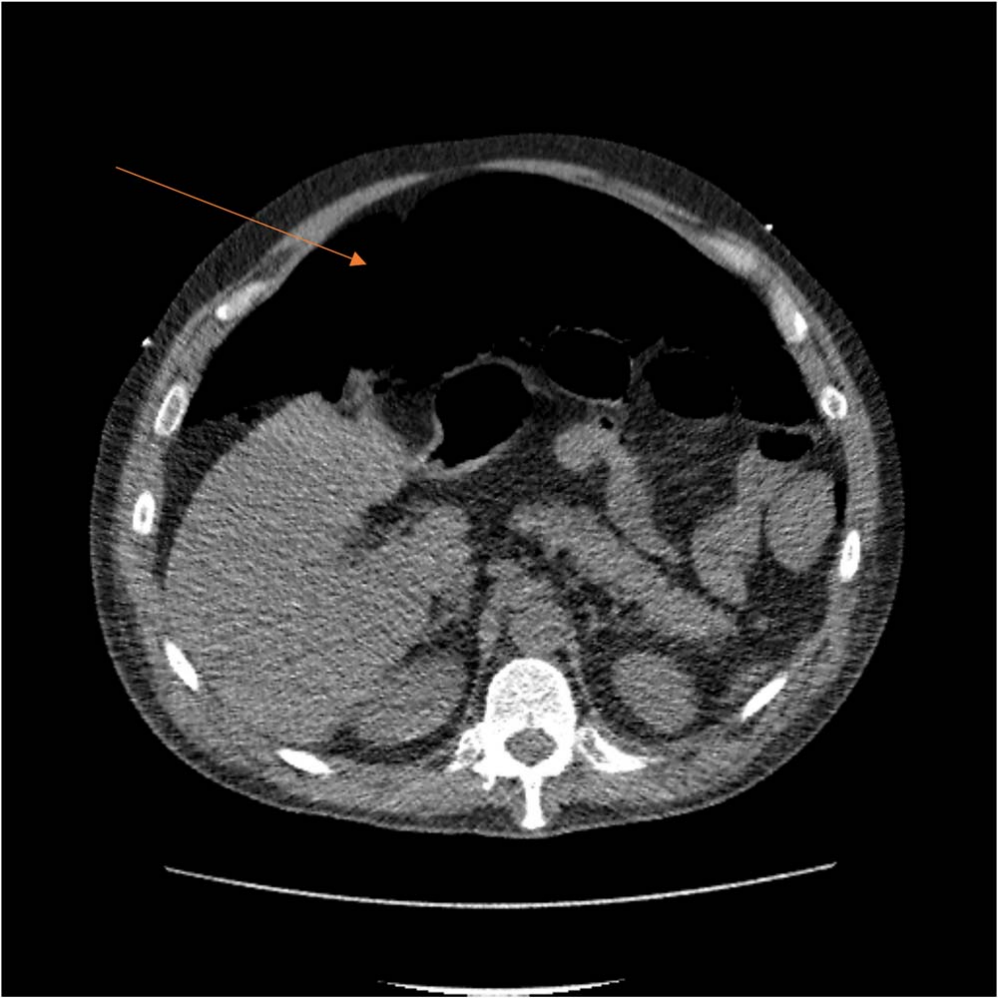
CT scan demonstrating pneumo-peritoneum (arrow), indicative of a hollow viscus perforation.

**Figure 6:**
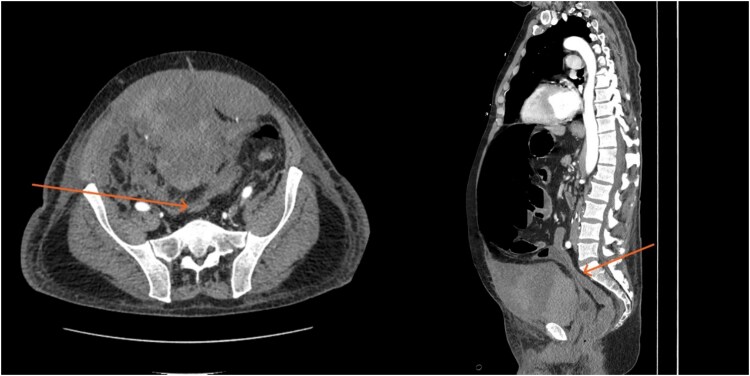
CT scan demonstrating compression of large bowel (arrows) by the enlarged hematoma.

By hospital day 6, the patient’s condition improved. He was weaned from mechanical ventilation, his renal function improved (creatinine declined to 0.7 mg/dl), and hemoglobin stabilized at 8.4 gm/dl. The patient was clinically stable, and on hospital day 8, he was set up for discharge to a rehabilitation institution. However, on this day, he developed generalized abdominal pain associated with tachycardia, pulse = 118, and leukocytosis (white blood cells = 14.8 × 10^9^/l). Physical exam revealed a distended abdomen with diffuse tenderness and sluggish intestinal sounds. A CT scan showed a large volume of pneumoperitoneum, minimal free fluid, and mildly dilated small bowel loops, consistent with a hollow viscus perforation with no identifiable source of perforation (Fig. 5) and a compression of rectosigmoid region against sacral promontory by the enlarged hematoma (Fig. 6). The right colon was decompressed compared with the prior study, and the RSH size remained unchanged, but there was progression of VTE burden. Prior to the acute deterioration, the patient had intermittently reported mild abdominal discomfort, which was initially attributed to the known RSH. There were no clinical signs suggestive of bowel obstruction, such as vomiting or significant abdominal distension. While the patient noted some degree of constipation, he reported that this was consistent with his baseline bowel habits and did not raise concern for new pathology.

The acute care surgery service was consulted for the CT findings, and operative intervention was recommended in the form of an exploratory laparotomy. Upon incision of the abdominal wall, approximately 500 cc of old clotted blood and liquified hematoma were encountered. The cecum was noted to be indurated and under external pressure; further exposure revealed there was an anterior hole proximally 0.5–1 cm. Additionally, it was noted that the RSH was compressing the lower sigmoid colon and upper rectum against sacral promontory, nearly occluding it. It was thought that, mechanistically, the back pressure on the rectum caused dilation of the cecum and subsequent perforation. Ischemic perforation due to thrombosis or impaired perfusion was also considered. However, this was deemed unlikely intraoperatively, as there were no signs of ischemia or necrosis in the surrounding bowel, and the remainder of the colon appeared well-perfused.

An ileocecectomy was performed, and due to the critical condition of the patient, an ABThera dressing was placed, and the patient was scheduled for a second-look laparotomy and closure of the abdomen the following day. On the second-look laparotomy, no additional pathology was elucidated, and it was decided that both an ileostomy and a mucous fistula were needed.

The patient had a stable post-operative period and was discharged nine days after his second-look laparotomy due to pulmonary-related complications.

## Discussion

RSH results from rupture of superior or inferior epigastric arteries or their branches inside the rectus sheath [1, 2, 5]. The increased use of anticoagulation has led to an increased incidence of RSH and, subsequently, a higher rate of its life-threatening complications [6]. Other predisposing factors include obesity, trauma, corticosteroid therapy, arterial hypertension, and previous abdominal surgeries [7].

It’s reported that mortality from RSH is 4% in the geriatric population and increases to 25% in the presence of anticoagulation [8].

RSH most commonly occurs in the infra-umbilical region, below the arcuate line, where the posterior rectus sheath is absent, and the epigastric vessels are relatively fixed and more vulnerable to injury [9].

As a result of this anatomical structure, RSH that arises from rupture of inferior epigastric artery tends to expand posteriorly and inferiorly with no restriction [10], which was the probable cause of the mass effect of RSH on the recto-sigmoid against the sacral promontory in our case.

In the literature, there are few cases of RSH causing mass effect leading to either obstruction or perforation [11–14], and to our knowledge, there is only one other case report published in 2012 that describes ileocecal perforation secondary to a rectus sheath hematoma [15].

When evaluating for a RSH, CT scan is the investigation of choice, with sensitivity and specificity of 100% [2].

Most RSH are managed conservatively, with treatment modalities that consist of reversing coagulopathy, bed rest, pain management, compression, and blood transfusions if needed [5].

This approach has a success rate reaching over 80% [9]. Operative or procedural intervention often depends on the patient’s clinical condition, degree of acute anemia, need for recurrent blood transfusion, and, most rarely, the occurrence of intra-abdominal complications.

Arteriography with selective embolization of the epigastric arteries is the first interventional line of intervention if needed [16], as open surgery may decrease the pressure inside the closed space of the rectus sheath, and worsening of the hemorrhage by decreasing the tamponade effect [17].

In many cases, RSH has a fairly benign course; however, this can develop into a serious condition and life-threatening condition, particularly in patients on anticoagulation therapy. Early diagnosis using a CT scan, careful follow-up, and close monitoring are essential. Consideration for invasive interventions, including selective embolization, may be necessary depending on the patient’s condition and the severity of the hematoma. Physicians should be aware of the potential for significant mass effects, such as ileocecal perforation.
